# Supplementary material for: MHC-I upregulation safeguards neoplastic T cells in the skin against NK cell-mediated eradication in mycosis fungoides
Source: Nat Commun. 2024 Jan 25;15:752. doi: 10.1038/s41467-024-45083-8 (PMC10810852; doi:10.1038/s41467-024-45083-8)
Supplement: Supplementary file 1 — Supplementary Information [file 41467_2024_45083_MOESM1_ESM.pdf]

Supplementary Tables

Supplementary Table 1. Patients' clinical characteristics

Sample	Gender	Age at sample acquisition (years)	Date of sample acquisition	Sample type	Disease stage at sample acquisition	TNMB at sample acquisition	First appearance of skin problem	First diagnosis of CTCL	Disease stage at 5 years' follow-up/ death	TNMB at 5 years' follow-up/death/SCT	Date of death
MF028_BE	Male	58	2014-06	blood	IA	T1N0M0B0	2012	2012-08	IIB	T3N0M0B0	2016-07
MF028_BL		60	2016-05	blood	IIB	T3N0M0B0	2012	2012-08	IIB	T3N0M0B0	2016-07
MF028_BLC		60	2016-06	blood	IIB	T3N0M0B0	2012	2012-08	IIB	T3N0M0B0	2016-07
MF028_SE		57	2013-05	skin	IA	T1N0M0B0	2012	2012-08	IIB	T3N0M0B0	2016-07
MF028_SL		60	2016-05	skin	IIB	T3N0M0B0	2012	2012-08	IIB	T3N0M0B0	2016-07
MF032_BE	Male	65	2014-02	blood	IIA	T2N1M0B1	2003	2011-05	IVA1	T3N0M0B2	Alive[a]
MF032_BL		67	2016-09	blood	IIB	T3N1M0B1	2003	2011-05	IVA1	T3N0M0B2	Alive[a]
MF032_SE		66	2014-09	skin	IIA	T2N1M0B1	2003	2011-05	IVA1	T3N0M0B2	Alive[a]
MF032_SL		68	2016-12	skin	IIB	T3N1M0B1	2003	2011-05	IVA1	T3N0M0B2	Alive[a]
MF030_BE	Male	68	2013-06	blood	IA	T1N0M0B0	2011	2011	IVB	T3N0M1B2	2018-12
MF030_BL		71	2016-11	blood	IIB	T3N0M0B0	2011	2011	IVB	T3N0M1B2	2018-12
MF030_SE		68	2013-06	skin	IA	T1N0M0B0	2011	2011	IVB	T3N0M1B2	2018-12
MF030_SL		71	2016-09	skin	IIB	T3N0M0B0	2011	2011	IVB	T3N0M1B2	2018-12
MF029_S	Female	71	2013-03	skin	IB	T2N0M0B0	2006	2009	IVB	T3N1B0bM1	2014-12
MF040_S	Male	66	2014-02	skin	IA	T1N0M0B0	2011	2013	IIB	T3N0B0M0	Alive[a, b]
MF035_S	Female	65	2013-09	skin	IB	T2N0M0B0	2003	2011-09	IVB	T3N0M1B0	2019-07
MF201_S	Female	70	2014-03	skin	IIA	T2N1M0B1	2008	2010-07	IVA1	T4N1M0B2	2014-10
MF200_S	Male	72	2014-02	skin	IIA	T2N1M0B1	2011	2013-10	IVA1	T4N0M0B2	Alive[a]
MF202_S	Male	76	2013-06	skin	IIA	T2N2M0B1	2010	2010-12	IVA2	T4N3M0B2	2013-08
MF053_S	Male	54	2014-10	skin	IB	T2N0M0B0	2014	2014-08	IVA1	T3N2M0B2	2015-10
MF056_S	Male	48	2017-02	skin	IB	T2N0M0B0	2005	2011	IB	T2N0M0B0	Alive[a]
MF057_S	Female	66	2017-01	skin	IB	T2N0M0B0	2011	2016-12	IB	T2N0M0B0	Alive[a]
MF042_S	Male	77	2013-03	skin	IB	T2N0M0B0	2004	2013	IIB	T3N0M0B0	Alive[a]
MF046_S	Female	73	2015-10	skin	IB	T2N0M0B0	2009	2015-03	IB	T2N0M0B0	2018-01

CTCL, cutaneous T cell lymphoma; SCT, stem cell transplantation; TNMB, tumor-node-metastasis-blood.

[a]As of August 26, 2023.

[b]Transplantation.

Supplementary Table 2. FastQC analysis

Sample	# cells that passed FastQC control	# cells with TCR alpha or TCR β	# cells with TCR alpha chain	# cells with β chain	# single bystanders	# bystander groups	# main clone	# related to main clone
MF028_BE	68	48	20	38	45	3	0	0
MF028_BL	81	72	47	68	35	36	1	0
MF028_BLC	86	59	35	50	15	20	19	5
MF028_SE	85	68	31	59	29	0	39	0
MF028_SL	75	45	13	44	2	0	43	0
MF032_BE	93	62	18	58	6	0	56	0
MF032_BL	85	72	40	66	22	14	36	0
MF032_SE	93	80	67	78	16	4	58	2
MF032_SL	83	78	59	74	27	8	39	4
MF030_BE	71	53	26	38	30	19	4	0
MF030_BL	89	70	11	67	4	0	66	0
MF030_SE	90	73	47	70	30	0	37	6
MF030_SL	51	38	21	37	14	0	23	1
MF029_S	88	85	60	82	6	2	56	21
MF040_S	4	0	0	0	0	0	0	0
MF035_S	50	34	21	24	6	7	20	1
MF201_S	96	95	88	95	7	0	75	13
MF200_S	96	96	93	95	6	6	25	59
MF202_S	59	56	16	55	2	0	41	13
MF053_S	96	37	8	31	8	15	13	1
MF056_S	51	28	17	21	6	9	12	1
MF057_S	27	15	12	9	2	2	6	5
MF042_S	95	90	74	82	3	5	56	26
MF046_S	39	17	16	13	0	0	17	0

TCR, T cell receptor.

Supplementary Table 3. Computational vs. flow cytometry analysis of malignant clones

	Computationally reconstructed clones: α chain	Computationally reconstructed clones: β chain	Corresponding TCR Vβ segments as detected computationally*	Corresponding TCR Vβ protein chain as detected by flow cytometry*
**Sample**	**Malignant TCR α chain**	**Malignant β chain**	**TRBC**	**Vβ**
MF028_SE	TRAV26-1_AGTCGTTAGTG_TRAJ53	TRBV20-1_TAGAGGACTAGCGGGCTCCT_TRBJ2-1	TRBV20-1	Vβ2
MF028_SL	TRAV26-1_AGTCGTTAGTG_TRAJ53	TRBV20-1_TAGAGGACTAGCGGGCTCCT_TRBJ2-1	TRBV20-1	Vβ2
MF032_SE	TRAV6_TGTGCCGTACGGGATA_TRAJ12	TRBV4-1_CCAAGCCCTCGGGGGGGATAAT_TRBJ1-6	TRBV4-1	Vβ7.1
MF032_SL	TRAV6_TGTGCCGTACGGGATA_TRAJ12	TRBV4-1_CCAAGCCCTCGGGGGGGATAAT_TRBJ1-6	TRBV4-1	Vβ7.1
MF030_SE	TRAV12-2_TGAACTGGGATA_TRAJ33	TRBV20-1_AGAGATTTAACTAGCGGGAGCTTCCCCTAC_TRBJ2-7	TRBV20-1	Vβ2
MF030_SL	TRAV12-2_TGAACTGGGATA_TRAJ33	TRBV20-1_AGAGATTTAACTAGCGGGAGCTTCCCCTAC_TRBJ2-7	TRBV20-1	Vβ2
MF029_S	TRAV29_DV5_CAAGCCGCACTAC_TRAJ40	TRBV20-1_GTGCTCCCCGACTAGCGGGAAGCTTACCCTAC_TRBJ2-7	TRBV20-1	Vβ2
MF040_S	-	-	-	-
MF035_S	TRAV29_DV5_AGCAAATTCA_TRAJ45	TRBV2_AGCAGCGGGGACAGACTCTACG_TRBJ2-7	TRBV2	Vβ22
MF201_S	TRAV24_CCTTTTCCTCA_TRAJ40	TRBV27_GTTTACTAGCGGGGGGGACAGA_TRBJ2-3	TRBV27	Vβ14
MF200_S	TRAV13-1_CAAGTCCGAACA_TRAJ30	TRBV20-1_CTAGACGGGACACCCCTAGCAC_TRBJ2-3	TRBV20-1	Vβ2
MF202_S	TRAV12-1_TGTGGCCCAGATTT_TRAJ23	TRBV5-1_CTTGGCTGGAGATA_TRBJ2-3	TRBV5-1	Vβ5.1
MF053_S	TRAV3_GAGAGCCCCCATAAGGAAT_TRAJ35	TRBV12-3_AGTTTCGGAATGACAGGGGGTTATTCA_TRBJ1-6	TRBV12-3	Vβ8
MF056_S	TRAV17_ACGGAGCCCCTTTATG_TRAJ42	TRBV12-3_CAGTTCACCAGGGGTCAGCAC_TRBJ2-3	TRBV12-3	Vβ8
MF057_S	TRAV6_GTGCTCCATGGGGGTTACATGGCT_TRAJ37	TRBV18_CACCAAATAGCAAGTCTGG_TRBJ1-3	TRBV18	-
MF042_S	TRAV41_GTCAGCCAATAT_TRAJ47	TRBV4-3_CAAGATGGGCCTAGTGGTGACAAT_TRBJ2-1	TRBV4-3	-
MF046_S	TRAV13-2_AGAGATTCCTTCGATGG_TRAJ12	TRBV2_AGCAGCCCTAAC_TRBJ1-2	TRBV2	-
Healthy T	-	-	-	-

TCR, T cell receptor
*According to the ImMunoGeneTics (IMGT), which serves as an established resource offering a standardized nomenclature for genes and alleles, encompassing diverse variable immune structures, such as TCRs.

Supplementary Table 4. Sequences of primers used in RT-qPCR analysis

Primer name	Sequence
**IL32_F**	5′- GAACTTTTGGCCGCCATGT-3′
**IL32_R**	5′- GGGCCTTCAGCTTCTTCATGT-3′
**HLA-A_F**	5′-AGATACACCTGCCATGTGCAGC-3′
**HLA-A_R**	5′- GATCACAGCTCCAAGGAGAACC-3′
**HLA-B_F**	5′-CTGCTGTGATGTGTAGGAGGAAG-3′
**HLA-B_R**	5′ -GCTGTGAGAGACACATCAGAGC-3′
**HLA-C_F**	5′-GGAGACACAGAAGTACAAGCGC-3′
**HLA-C_R**	5′- ACATCCTCTGGAGGGTGTGAGA-3′
**ActB_F**	5′- AGCCTCGCCTTTGCCGA-3′
**ActB_R**	5′- CTGGTGCCTGGGGCG-3′

RT-qPCR, reverse transcription quantitative real-time polymerase chain reaction.

Supplementary Figure 1

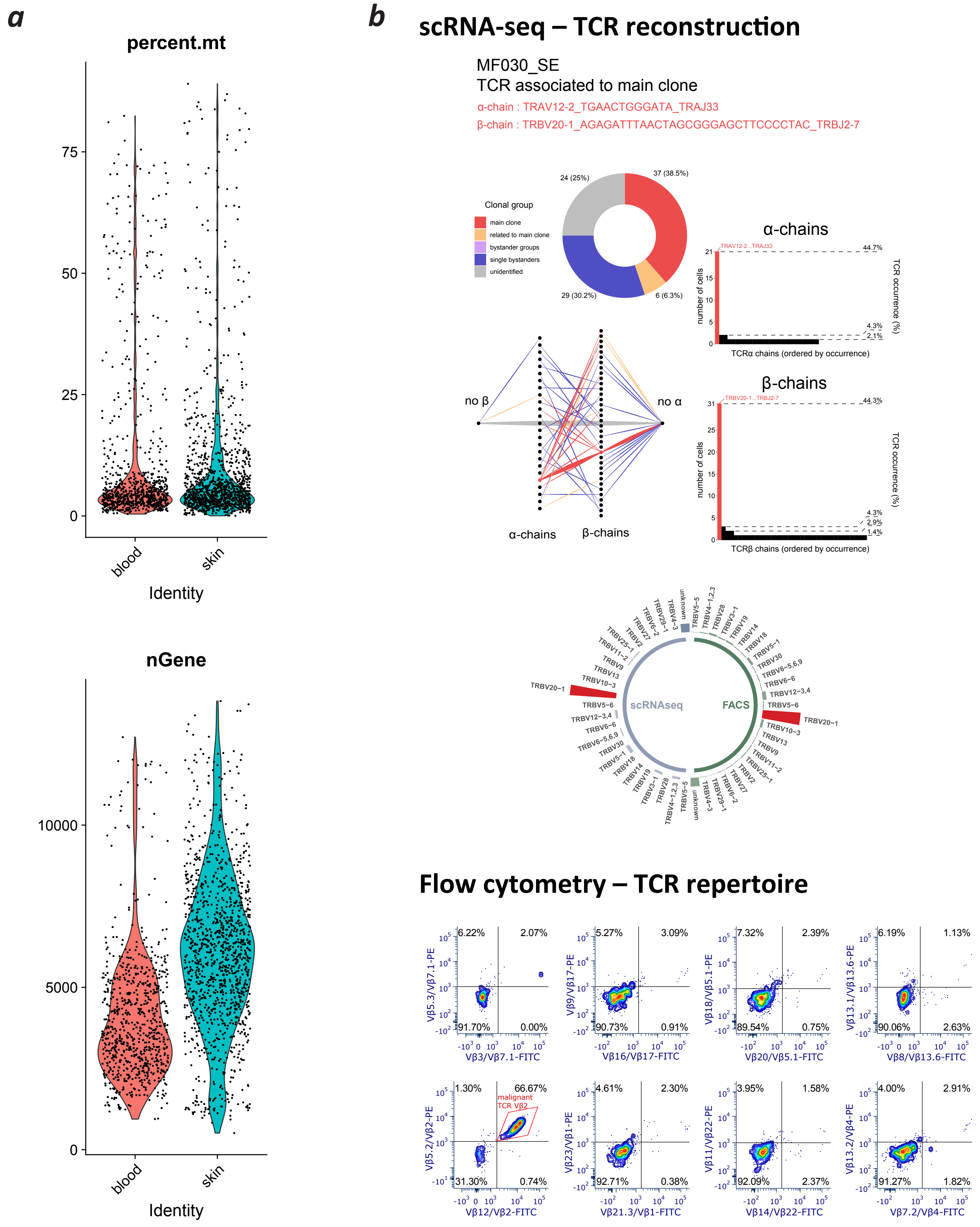

Supplementary Fig. 1 | The quality of T cells, comparison of malignant clonal T-cell identification and classification of T cells from each individual patient.

a, The T cells (N=573 for blood and N=1174 for skin) that passed FastQC control had low mitochondrial cDNA content and high numbers of alignable expressed genes. **b**, Comparison of malignant clonal T-cell identification between computational TCR clonality reconstruction based on scRNA-seq data (clonogram) and flow cytometry (representative example of N=10 patients). Clonality rules and classification of T cells from each individual patient into two clone-related populations, ‘main-clone’ and ‘related-to-main-clone’, and two nonclonal bystander populations, ‘bystander groups’ and ‘single bystanders’.

Supplementary Figure 2 (part 1)

MF028_SE

TCR associated to main clone

α-chain : TRAV26-1_AGTCGTTAGTG_TRAJ53

β-chain : TRBV20-1_TAGAGGACTAGCGGGCTCCT_TRBJ2-1

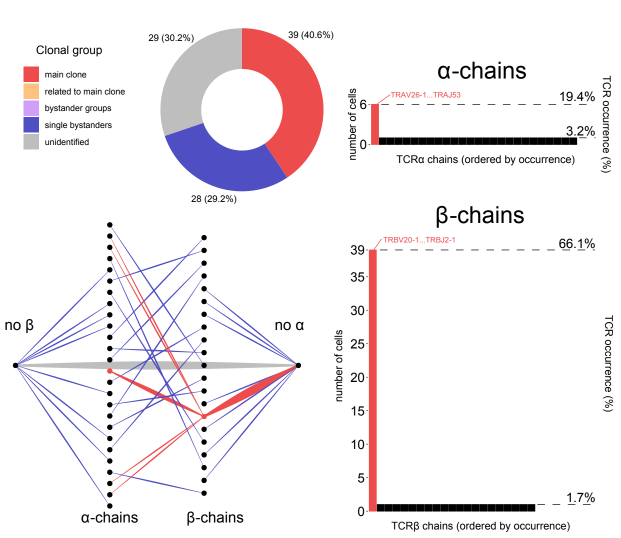

MF028_SL

TCR associated to main clone

α-chain : TRAV26-1_AGTCGTTAGTG_TRAJ53

β-chain : TRBV20-1_TAGAGGACTAGCGGGCTCCT_TRBJ2-1

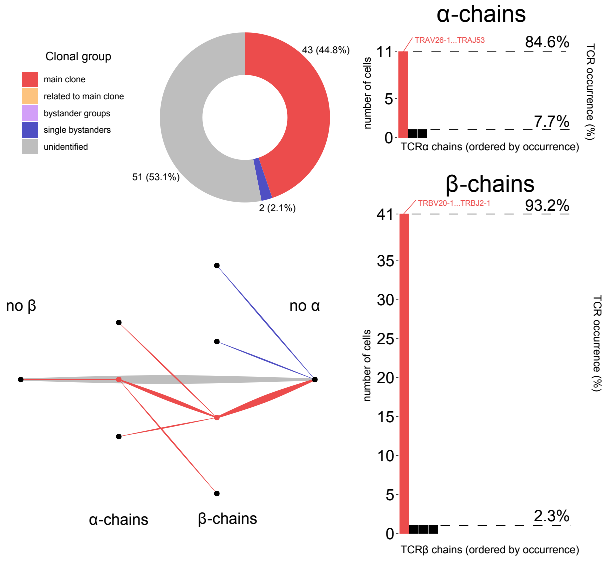

MF032_SE

TCR associated to main clone

α-chain : TRAV6_TGTGCCGTACGGGATA_TRAJ12

β-chain : TRBV4-1_CCAAGCCCTCGGGGGGGATAAT_TRBJ1-6

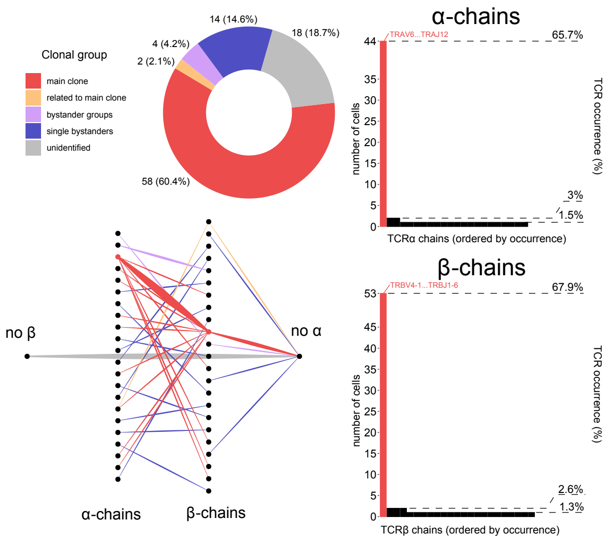

MF032_SL

TCR associated to main clone

α-chain : TRAV6_TGTGCCGTACGGGATA_TRAJ12

β-chain : TRBV4-1_CCAAGCCCTCGGGGGGGATAAT_TRBJ1-6

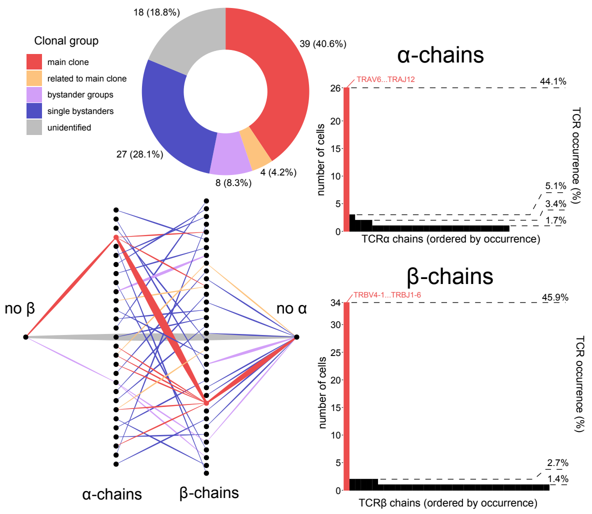

Supplementary Figure 2 (part 2)

MF030_SE
TCR associated to main clone
α-chain : TRAV12-2_TGAACTGGGATA_TRAJ33

β-chain : TRBV20-1_AGAGATTTAACTAGCGGGAGCTTCCCCTAC_TRBJ2-7

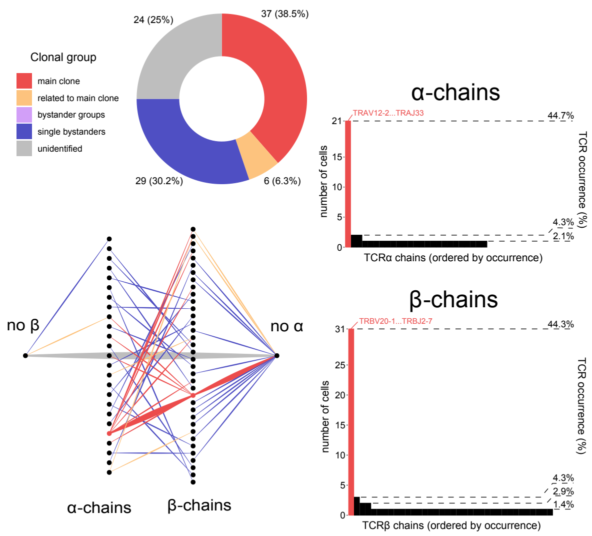

MF030_SL
TCR associated to main clone
α-chain : TRAV12-2_TGAACTGGGATA_TRAJ33

β-chain : TRBV20-1_AGAGATTTAACTAGCGGGAGCTTCCCCTAC_TRBJ2-7

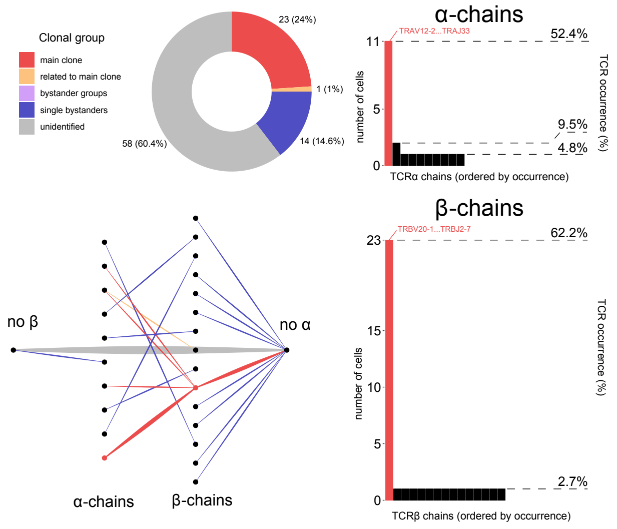

MF029
TCR associated to main clone
α-chain : TRAV29_DV5_CAAGCCGCACTAC_TRAJ40

β-chain : TRBV20-1_GTGCTCCCCGACTAGCGGGAAGCTTACCCTAC_TRBJ2-7

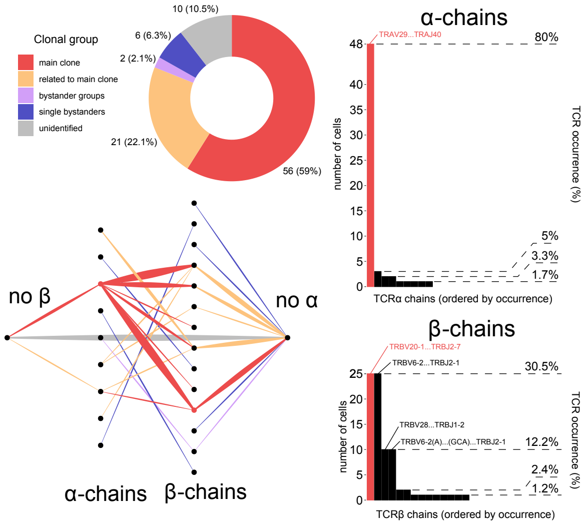

MF035
TCR associated to main clone
α-chain : TRAV29_DV5_AGCAAATTCA_TRAJ45

β-chain : TRBV2_AGCAGCGGGGACAGACTCTACG_TRBJ2-7

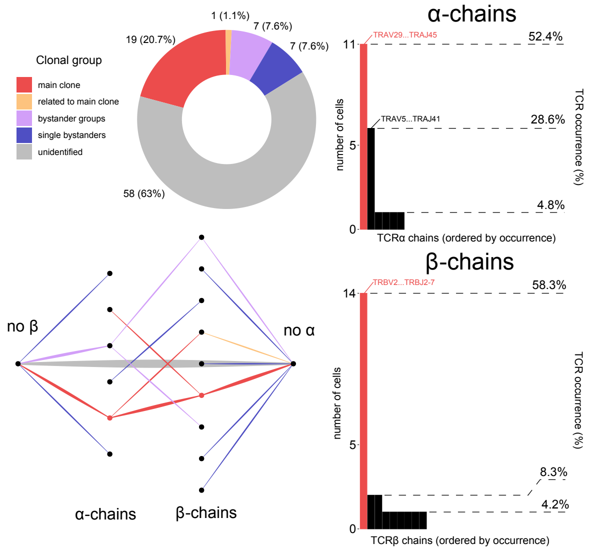

Supplementary Figure 2 (part 3)

MF201
TCR associated to main clone

α-chain : TRAV24_CCTTTTCCTCA_TRAJ40

β-chain : TRBV27_GTTTACTAGCGGGGGGGACAGA_TRBJ2-3

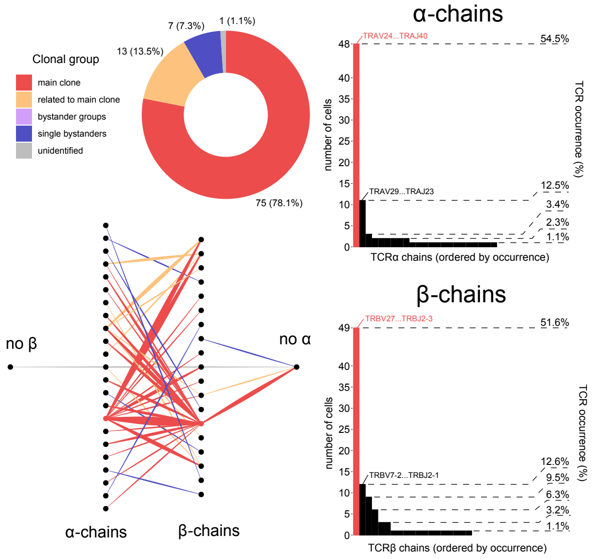

MF200
TCR associated to main clone

α-chain : TRAV13-1_CAAGTCCGAACA_TRAJ30

β-chain : TRBV20-1_CTAGACGGGACACCCCTAGCAC_TRBJ2-3

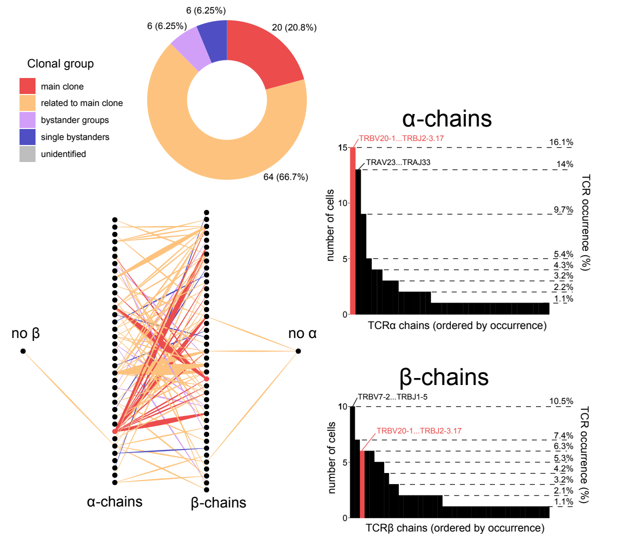

MF202
TCR associated to main clone

α-chain : TRAV12-1_TGTGGCCCAGATTT_TRAJ23

β-chain : TRBV5-1_CTTGGCTGGAGATA_TRBJ2-3

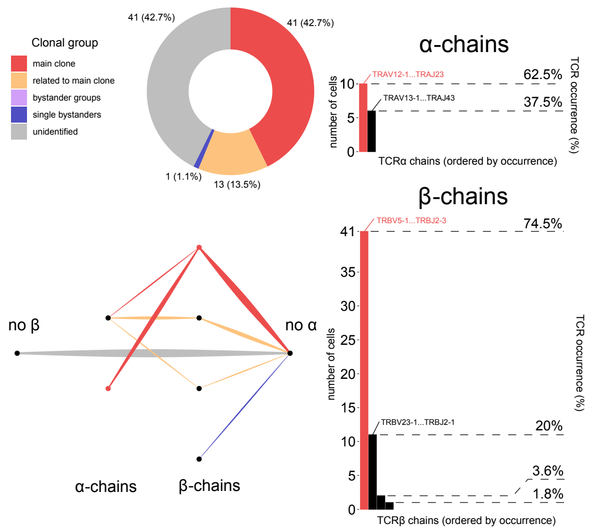

MF053
TCR associated to main clone

α-chain : TRAV3_GAGAGCCCCCATAAGGAAT_TRAJ35

β-chain : TRBV12-3_AGTTTCGGAATGACAGGGGGTTATTCA_TRBJ1-6

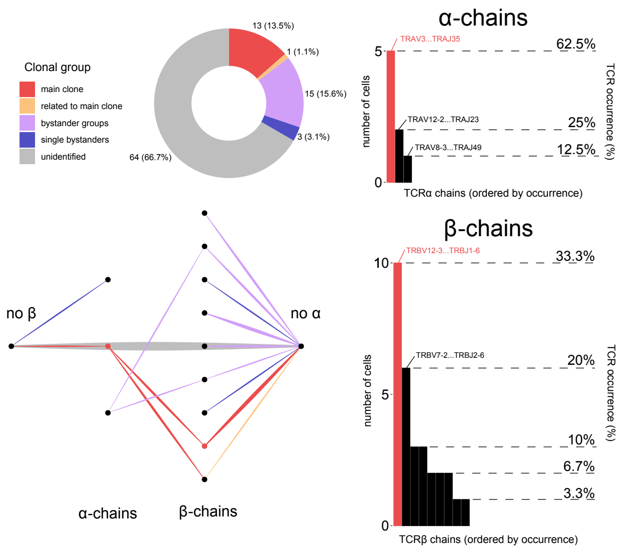

Supplementary Figure 2 (part 4)

MF056
TCR associated to main clone
α-chain : TRAV17_ACGGAGCCCCTTTATG_TRAJ42
β-chain : TRBV12-3_CAGTTCACCAGGGGTCAGCAC_TRBJ2-3

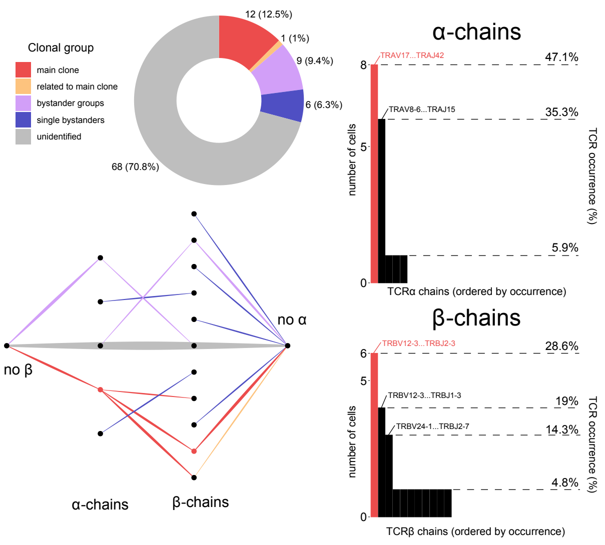

MF057
TCR associated to main clone
α-chain : TRAV6_GTGCTCCATGGGGGTTACATGGCT_TRAJ37
β-chain : TRBV18_CACCAAATAGCAAGTCTGG_TRBJ1-3

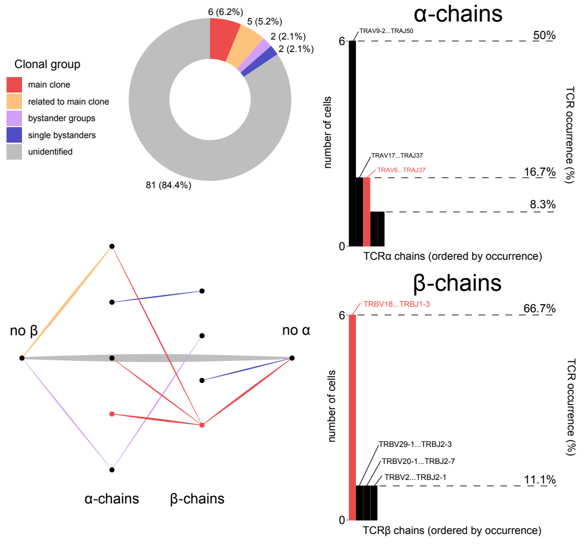

MF042
TCR associated to main clone
α-chain : TRAV41_GTCAGCCAATAT_TRAJ47
β-chain : TRBV4-3_CAAGATGGGCCTAGTGGTGACAAT_TRBJ2-1

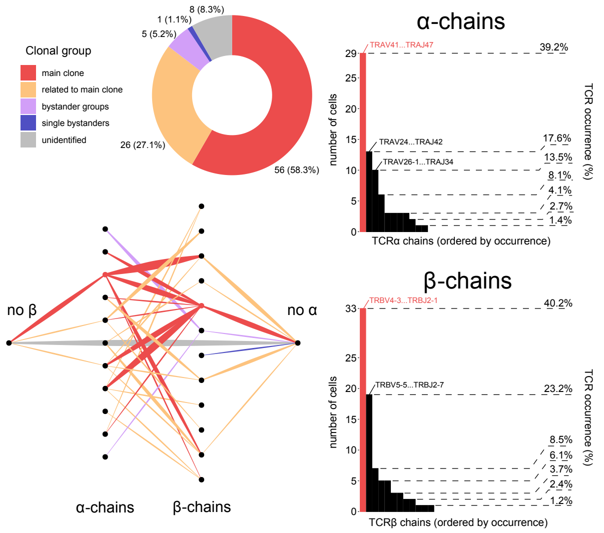

MF046
TCR associated to main clone
α-chain : TRAV13-2_AGAGATTCCTTCGATGG_TRAJ12
β-chain : TRBV2_AGCAGCCCTAAC_TRBJ1-2

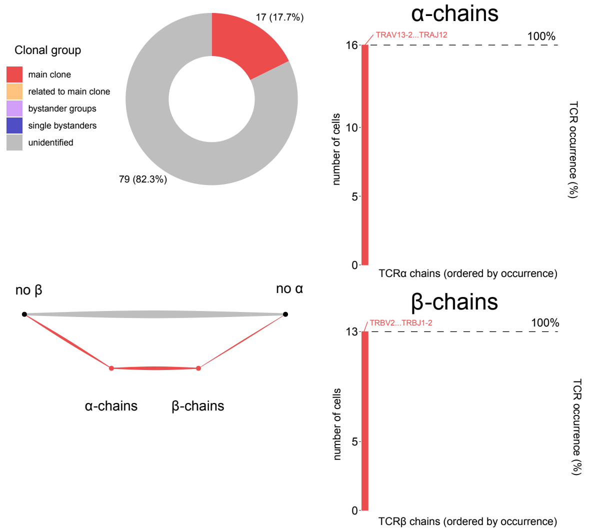

Supplementary Fig. 2 | T cell clonality assessment for each individual patient and respective skin lesion.

TCR sequence of the main clone (top), At the top of the figure, the TCR α- and β-chain sequences of the main (malignant) clone for each patient is noted. **Pie chart (middle),** In the middle of the figure, T cells from each patient are categorized into four groups based on clonality rules: "Main-Clone" in red- the largest group of T cells sharing the same combination of α and β chains; "Related-to-Main-Clone" in yellow - cells that did not match the α or β chains of the "main-clone" but had a relative association with "main-clone"; "Bystander Groups" in violet - cells with reconstructed chains that neither matched nor had any relative associations with the α or β chains of the "main clone". These cells needed to exhibit at least two cells sharing the same α and β chains within the sample; "Single Bystanders" in blue - cells lacking reconstructed α or β chains shared with any other cells in the sample. The pie chart visually represents the percentage of each of these populations, along with an "Unidentified" category in grey. **Histogram of TCR α-chain and β-chain (right),** The histograms of α-chain and β-chain depict the distributions of each reconstructed TCR α- and β- chain. On the x-axis, each column represents one reconstructed TCR α-chain (upper histogram) and one reconstructed TCR β-chain (bottom histogram) within the sample. The left y-axis indicates the actual number of cells having indicated TCR α-chain or TCR β-chain. On the right y-axis, the percentages indicate the occurrence of each TCR α-chain or TCR β-chain within the sample. **Clonogram (left bottom),** each node (black dot) indicates one individual TCR α-chain (left) or TCR β-chain (right). Each conencted edge (the connection between two nodes) represents a cell with reconstructed TCR α-chain and/or β-chain. The red connected edges connect the TCR α-chain and β-chain of the main (malignant) clone. The connected edge between α- and β-chain indicated one individual cell has that α- and β-chain. If the cell has only reconstructed TCR α- or β-chain, then the connected edge links to "no β" or "no α". The connected edge between "no β" and "no α" indicated the unidentified cell and is represented in grey colour. The thickness of the connected edge implies the number of the cells that share the same connection. The color of the connected edge corresponds to the T-cell classification.

Supplementary Figure 3

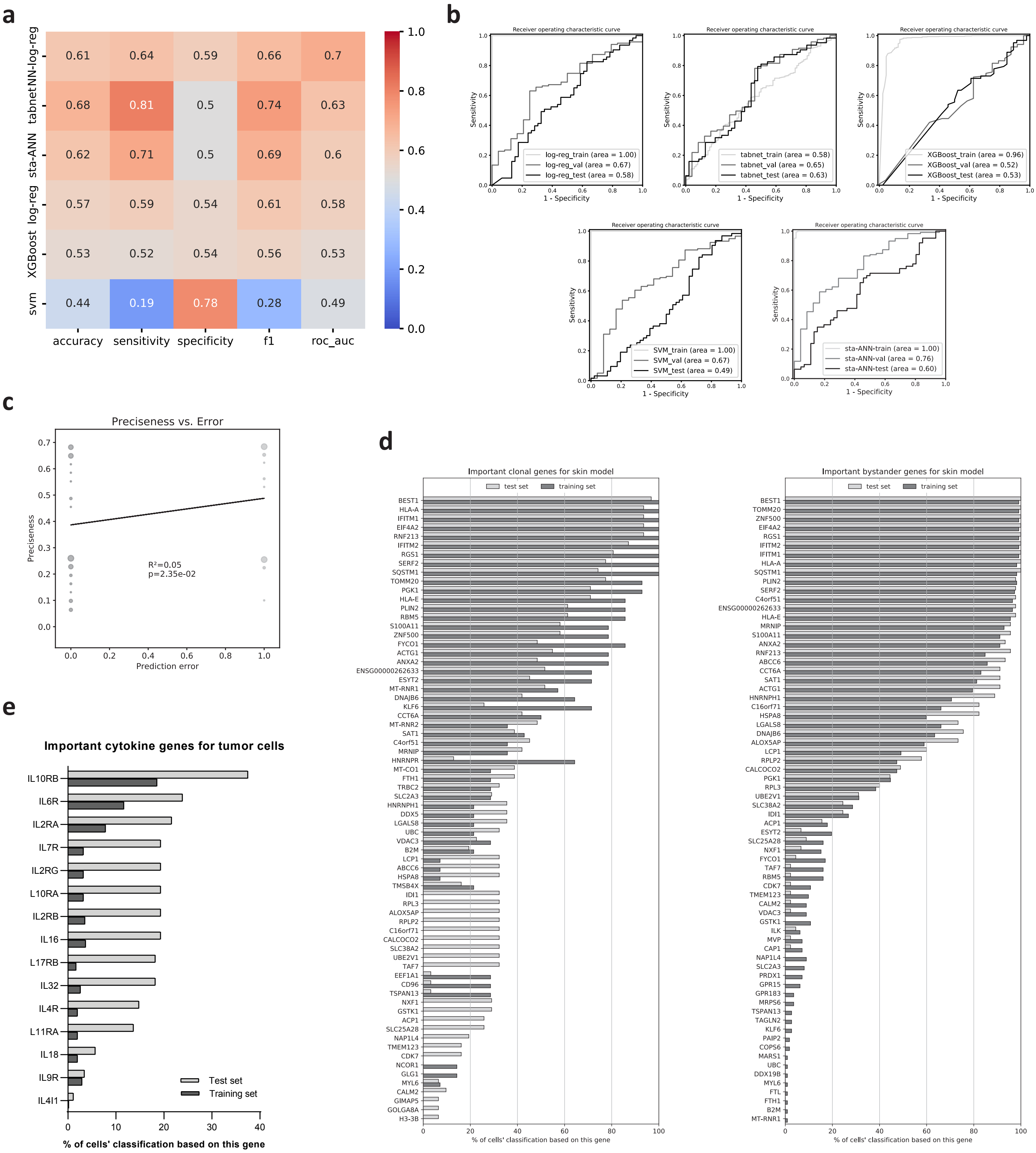

Supplementary Fig. 3 | Comparison of different artificial neural network methods and the most important genes for tumor-predicting.

a, The evaluation metrics of compared models trained on the skin T-cell model. Classification thresholds of different models were empirically determined using the validation set. **b**, The ROC of the log-reg, TabNet, XGBoost, SVM and standard-ANN method on skin in training, validation and test sets. **c**, Robustness of the NN-log-reg method. Preciseness (how close a prediction is to 0 or 1) was plotted against prediction error. The sizes of the circles represent the number of cells. Dark grey circles represent correctly classified cells, and light grey circles represent misclassified cells. The black line represents the trend line of all data points (linear regression). **d**, The most important genes for predicting whether a cell is a clonal (left panel) or bystander (right panel) cell, as predicted by an NN-log-reg model trained on skin cells only. **e**, The most important cytokine-related genes for identifying tumor T cells as predicted by an NN-log-reg model trained and tested on skin cells. The x axis indicates the percentage of cells for which the specific gene (on the y axis) is among these top 0.5 % in the 'feature importance' list. For instance, a value of 100 % for "gene A" means that "gene A" is among the top 0.5 % of 'important genes' for 100 % of correctly classified cells. N=667 for clone-related populations, 'main-clone' and 'related-to-main-clone', and N= 158 for nonclonal bystander populations, 'bystander groups' and 'single bystanders'.

Supplementary Figure 4

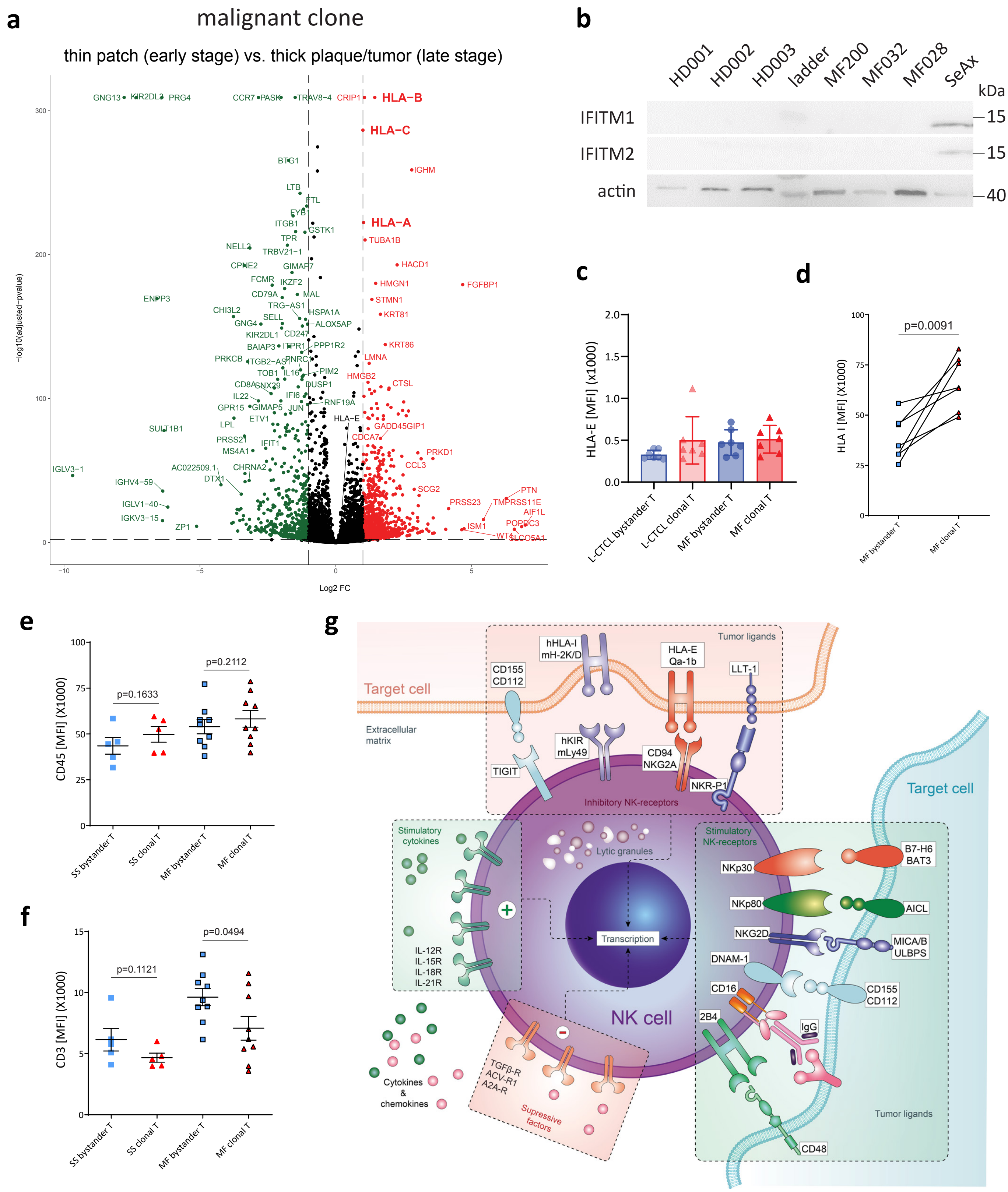

Supplementary Fig. 4 | MHC-I conceals tumor T cells in the skin from NK-cell surveillance

a, The scRNAseq analysis of dataset GSE173205 indicated the significant upregulation of classical MHC-I - HLA-A, HLA-B and HLA-C – in the tumor T cells from late-stage MF skin lesions (N=3). **b,** Western blot analysis demonstrating that IFITM1 and IFITM2 were barely detectable at the protein level on T cells from MF skin lesions (N=3) and healthy skin lesions (N=3). **c,** The expression of non-classical MHC-I (HLA-E) was generally low, with no significant statistical difference observed between the samples (N=7). Notably, higher HLA-E expression was detected only in the circulating T-cell tumors from one CTCL patient. Data were presented as mean values +/- SEM. The statistical differences were calculated using paired, two-tailed student's t test. **d**, Pair-wise comparison of MHC-I MFI in bystander and malignant skin T cells from patients with MF (N=7). Each color-indexed pair represents a dataset from an individual patient with MF. The p-value was calculated using paired, two-tailed student's t test. **e**, No difference in CD45 expression between malignant and bystander skin T cells of patients with MF (N=9) and blood T cells of patients with L-CTCL (N=5). Data were presented as mean values +/- SEM. The p-values were calculated using paired, two-tailed student's t test. **f**, Tendency toward decreased CD3 expression on malignant skin (N=9) and blood (N=5) T cells of patients with CTCL. Data were presented as mean values +/- SEM. The p-values were calculated using paired, two-tailed student's t test. **g**, Schematic representation of NK cell receptors and their ligands. NK cell activation is mediated by the integration of multiple activating and inhibitory signals. The schematic representation is created by co-author O.P.. Source data are provided as a Source Data file.

Supplementary Figure 5

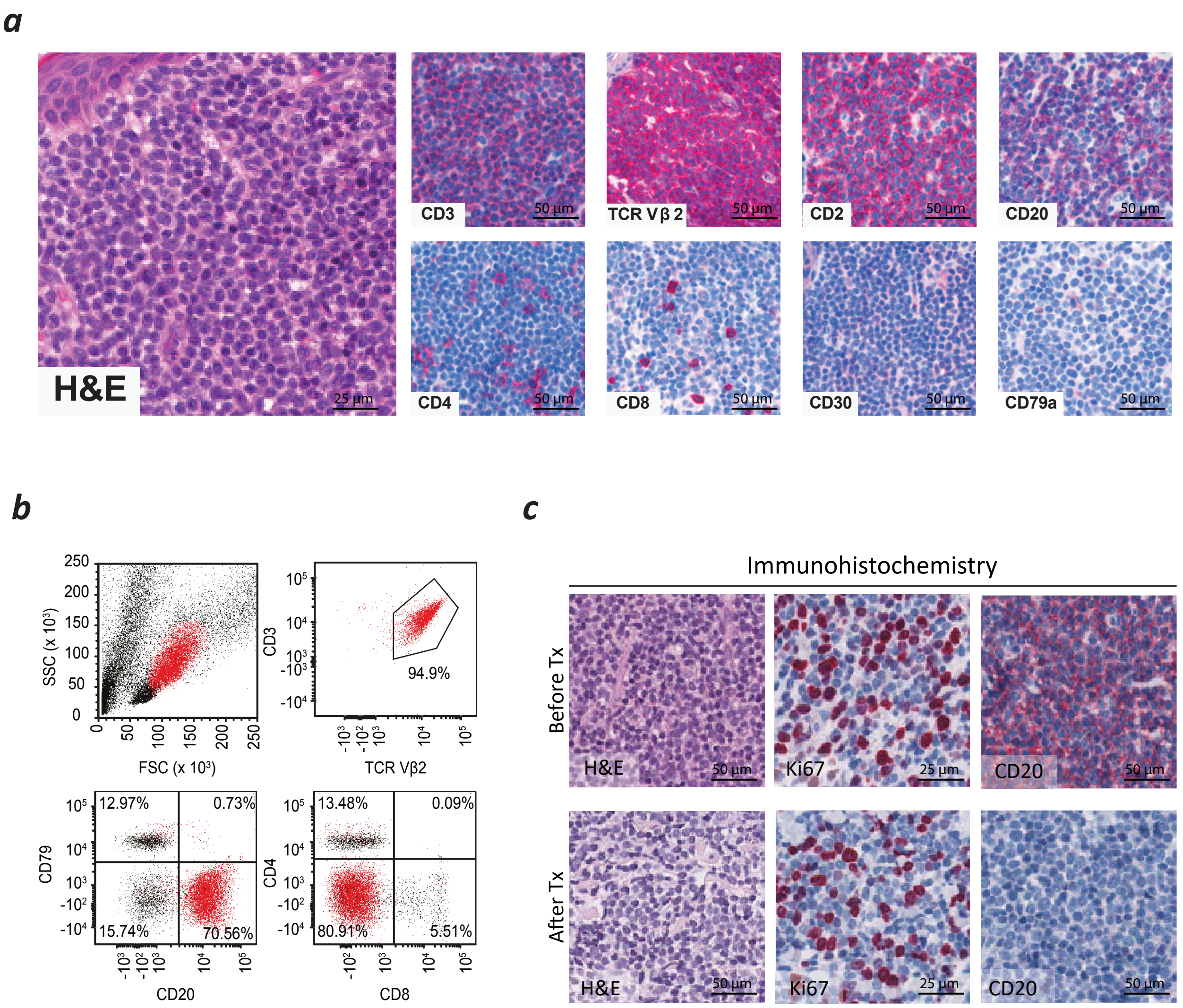

Supplementary Fig. 5 | Anti-CD20-mAb treatment for an aberrant $CD20^+$ MF.

a, Aberrant expression of CD20 by malignant T cells (N=1). The phenotype of MF tumor cells in skin lesions was $CD3^+$ TCR $V\beta2^+$ $CD2^+$ $CD4^-$ $CD8^-$ $CD30^-$ $CD20^+$ $CD79a^-$. **b**, Conclusive identification of the clonal malignant T-cell population (red dots) through expression of the TCR Vβ2 subset. Flow cytometry of skin T cells further revealed the loss of CD4 and aberrant expression of the B cell-specific differentiation antigen CD20 on malignant T cells (N=1). **c**, Reduced CD20 expression on tumor cells during anti-CD20-mAb treatment (N=1).

Supplementary Figure 6

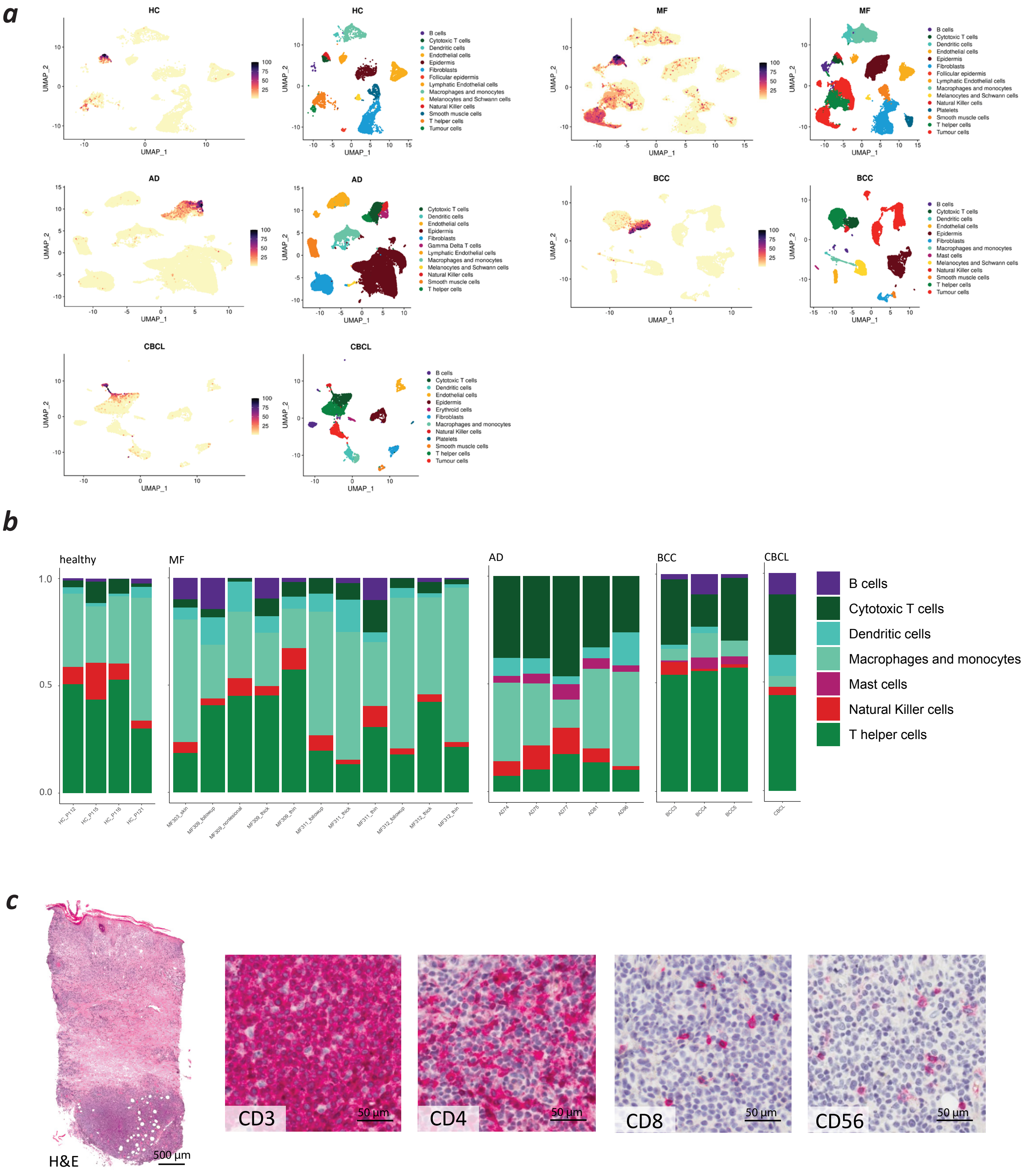

Supplementary Fig. 6 | The NK-cell proportion of healthy skin and skin cancers/disorders.

a, $CD56^+$ NK cells, key effector cells important for the effectiveness of tumor cell-surface antigen-targeted therapies, were present in typical MF skin lesions. **b,** Analysis of scRNAseq datasets on mycosis fungoides [MF: GSE173205 and GSE165623], atopic dermatitis [AD: GSE222840], basal cell carcinoma [BCC: GSE181907] and cutaneous B-cell lymphoma [CBCL: GSE173820]. The NK cells were identified by using the following gene signature: NKG7, KLRB1, KLRC1, KLRD1, KLRK1, CD7, GZMB, GNLY, NCAM1, GZMH, CCL4, IFNG, CCL4L2, FCGR3B and FCGR3A. **c**, $CD56^+$ NK cells, key effector cells important for the effectiveness of tumor cell-surface antigen-targeted therapies, were present in typical MF skin lesions (N=1).

Supplementary Figure 7

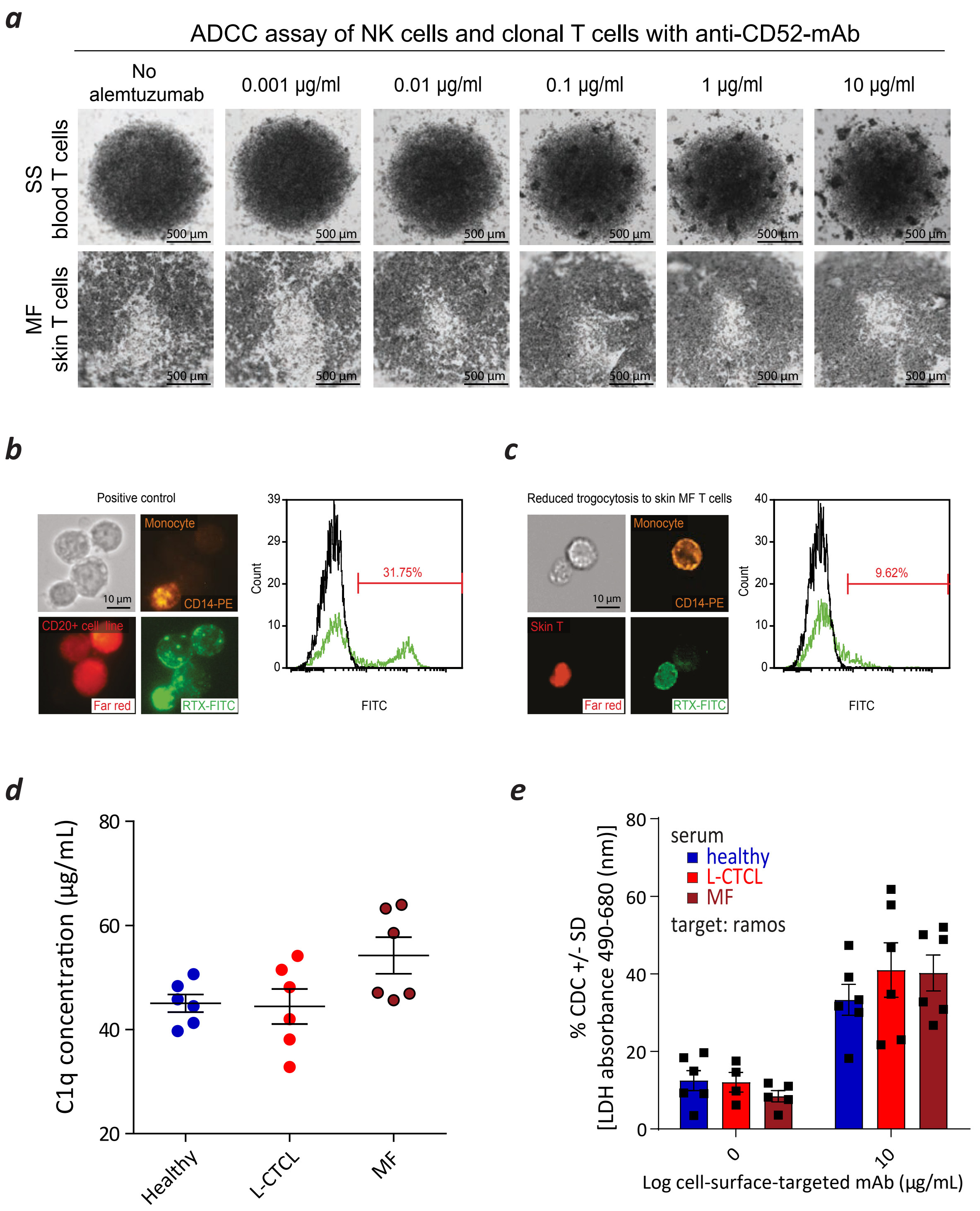

Supplementary Fig. 7 | Possible mechanisms of resistance to tumor-cell surface antigen targeted therapies in MF.

a, The presentation of ADCC clusters corresponds to functional ADCC activity. A greater number of visible clusters indicates stronger ADCC activity. The experiment was repeated for three times with similar results. **b-c**, Trogocytosis, a surface-molecule-transfer mechanism that can hamper ADCC, was ruled out as a possible mechanism for ineffective tumor cell-surface antigen-targeted therapy in MF. Trogocytosis served as a positive control by using Ramos cells as target cells (N=1). Low or reduced trogocytosis was observed using $CD20^{+}$ MF skin T cells as target cells (N=1). **d**, Serum concentrations of C1q, the key molecule in complement-dependent cytotoxicity, were similar between patients with CTCL (MF (N=6) and L-CTCL (N=6)) and healthy individuals (N=6). Data were presented as mean values +/- SEM. The statistical differences were calculated using unpaired, two-tailed student's t test. **e**, Patients with MF (N=6), L-CTCL (N=6) and healthy individuals (N=6) show similar serum levels of CDC activity. Data were presented as mean values +/- SEM. The statistical differences were calculated using unpaired, two-tailed student's t test. Source data are provided as a Source Data file.

Supplementary Figure 8

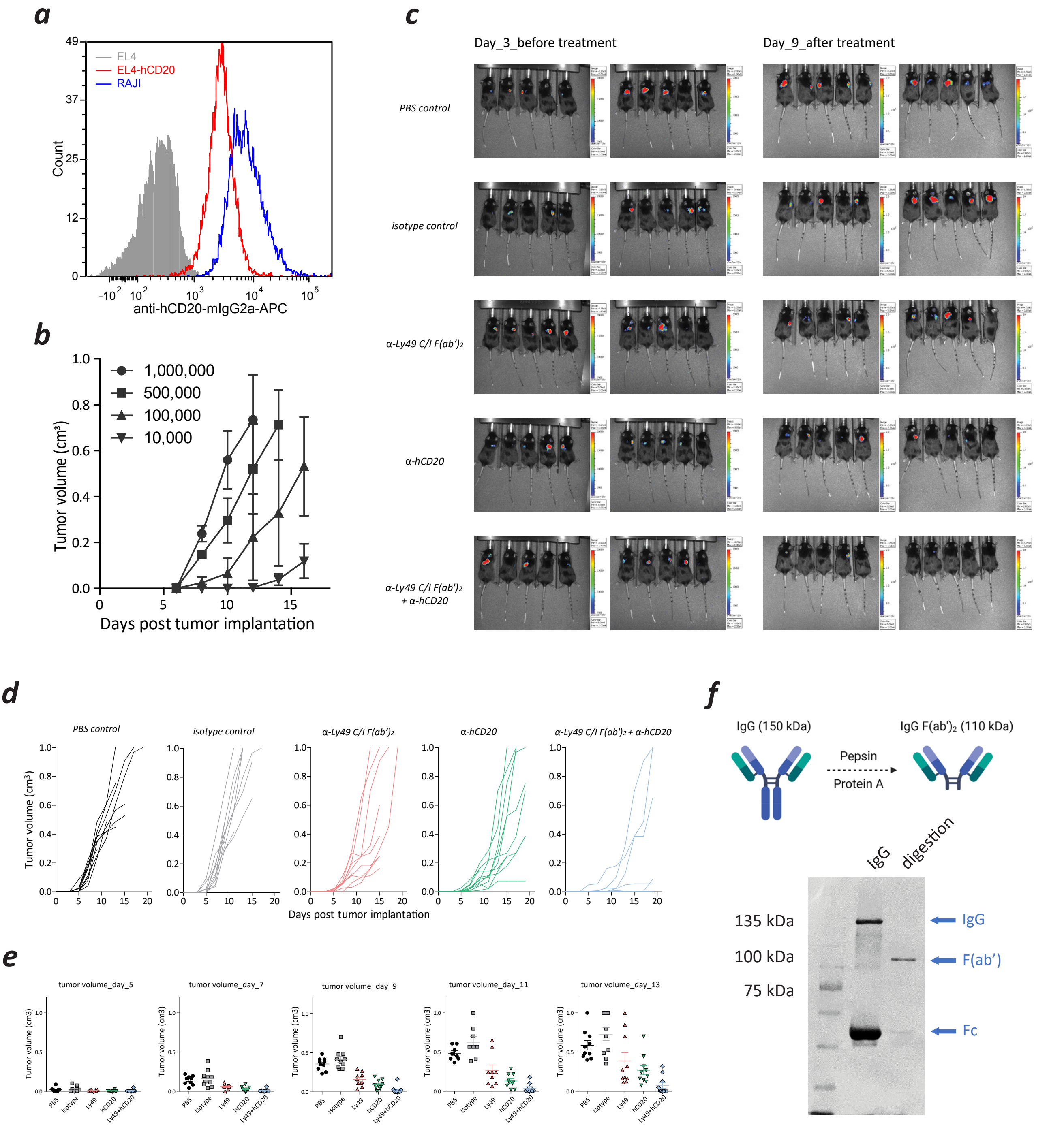

Supplementary Fig. 8 | In vivo studies of EL4-hCD20 murine model system.

a, EL4-hCD20 cells express human CD20 on the cell surface, as verified by APC-conjugated therapeutic anti-hCD20 mouse IgG2a. **b**, Varying numbers of EL4-hCD20 cells were implanted to determine the optimal number of tumor cells to use for in vivo studies (N=3 mice per group). Data were presented as mean values +/- SD **c**, IVIS results from the control, monotherapy and combined therapy groups before treatment on day 3 and after treatment on day 9 (N=10 mice per group)). **d**, Individual tumor volumes of EL4-hCD20 implants in the untreated (IgG control and PBS control), anti-Ly49 C/I F(ab')$_2$, anti-hCD20 and anti-Ly49 C/I F(ab')$_2$ + anti-hCD20 groups (N=10 mice per group). **e**, Plots showing the kinetics of tumor growth between the different groups (N=10 mice per group) of mice on day 5, day 7, day 9, day 11 and day 13 post-tumor transplantation. Data were presented as mean values +/- SEM. **f**, The scheme for preparing F(ab')$_2$ fragments from MHC-I-blocking IgG. The schematic representation is created with BioRender.com. The size of MHC-I-blocking IgG and its F(ab')$_2$ fragment after pepsin digestion and protein A purification, as verified by nonreducing SDS-PAGE. Source data are provided as a Source Data file.

Supplementary Figure 9

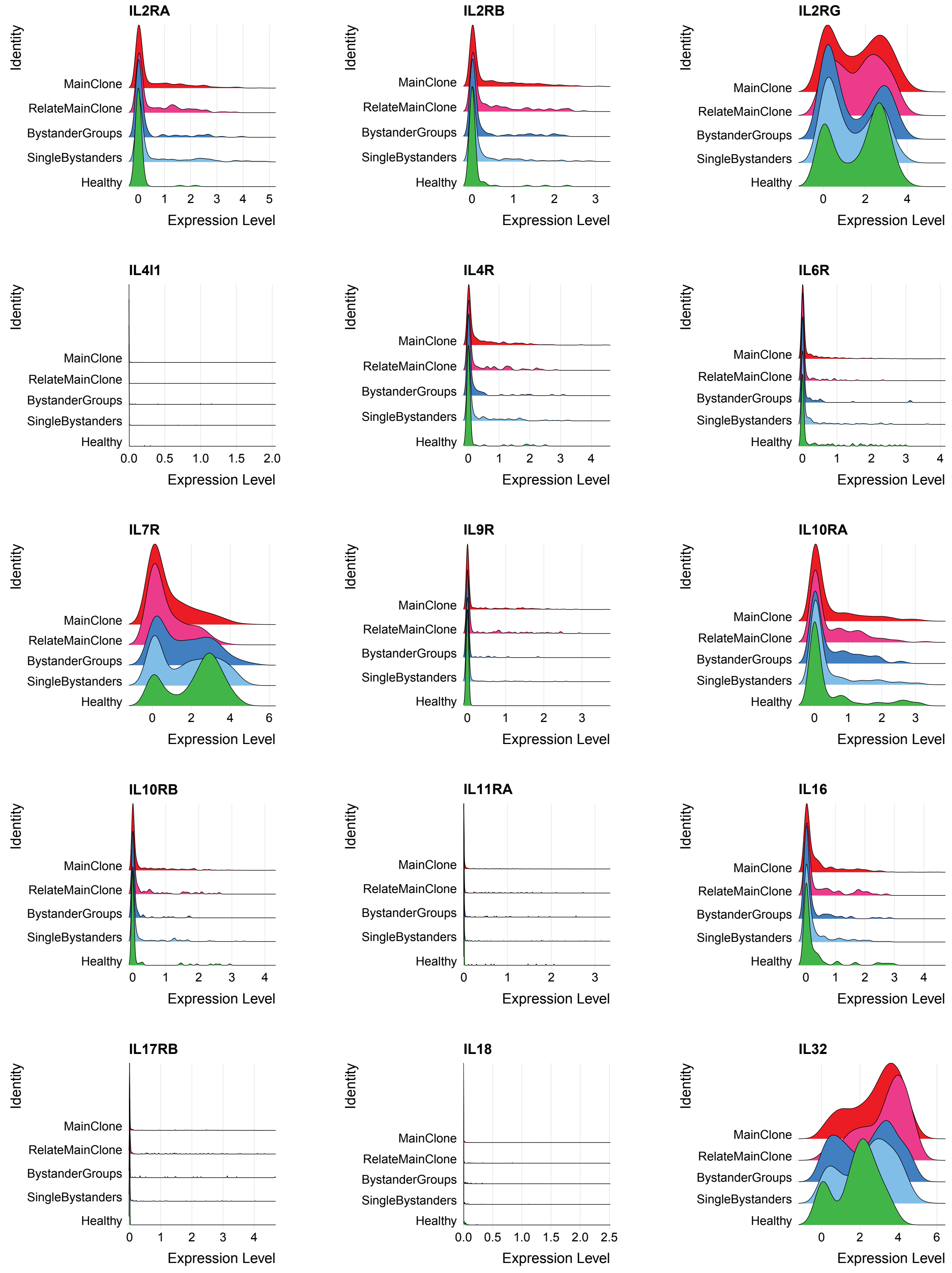

Supplementary Fig. 9 | Cytokine-related genes in malignant T cells

By analyzing the 15 important cytokine-related genes of malignant T cells that were recognized by our NN-log-reg method, malignant T-cell populations show significantly increased IL32 expression and decreased IL7R expression. N=526 for Main Clone; N=141 for Relate Main Clone; N=50 for Bystander Groups; N=108 for Single Bystanders; N=85 for Healthy.

Supplementary Figure 10

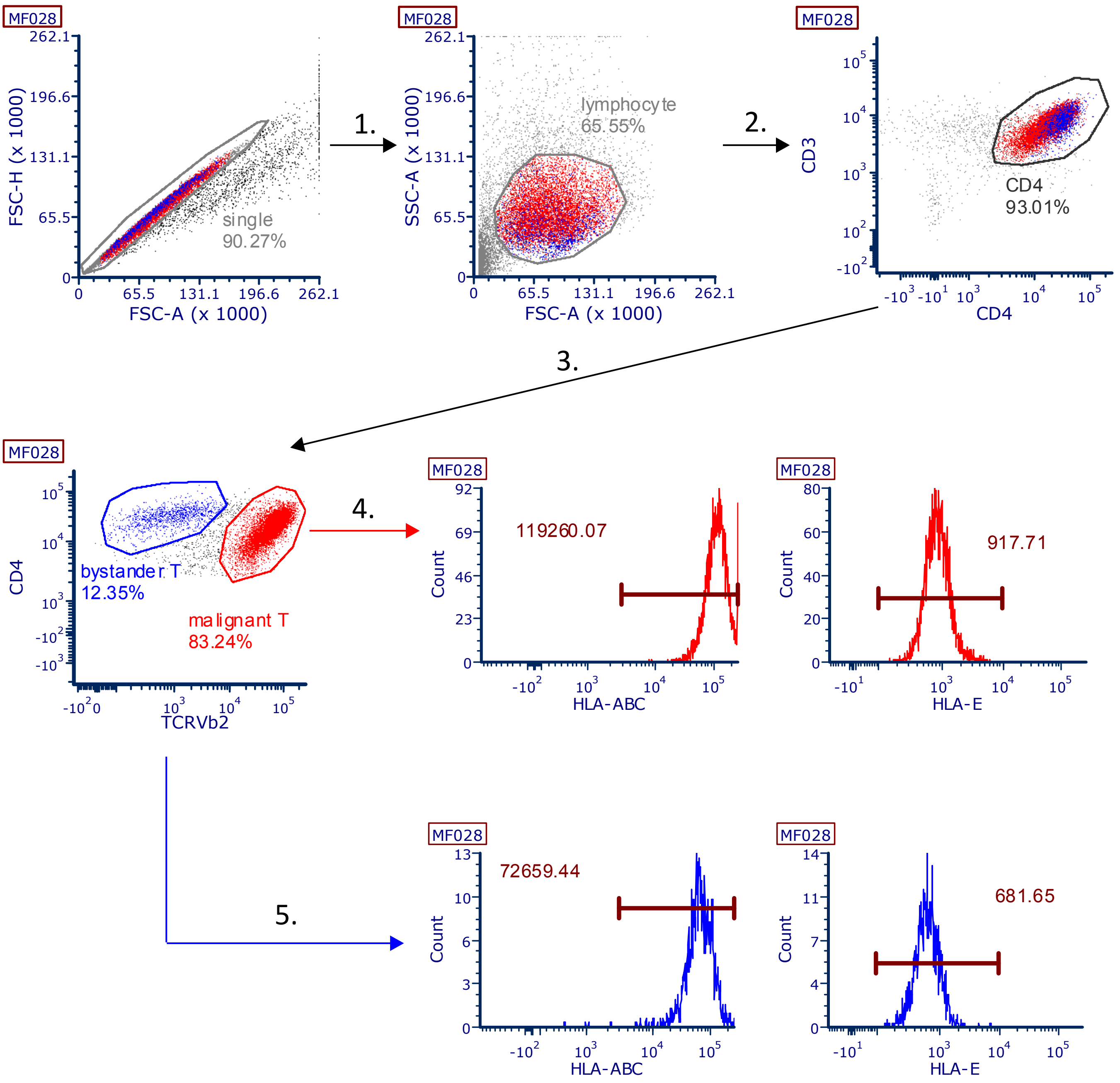

Supplementary Fig. 10 | Gating strategy for analyzing HLA-I and HLA-E expression on malignant and bystander T cells

Doublets were excluded using FSC-A and FSC-H. Lymphocyte population was gated by using FSC-A and SSC-A. Thereafter, $CD3^+/CD4^+$ T cells were gated, and malignant and bystander T cells were further separated based on patient-tumor-T-cell-specific TCR Vβ antibody for measuring mean fluorescence intensity (MFI) of HLA-I and HLA-E. FSC-A, forward scatter-area; FSC-H, forward scatter-height; SSC-A, side scatter-area.
