# Supplementary material for: MHC-I upregulation safeguards neoplastic T cells in the skin against NK cell-mediated eradication in mycosis fungoides
Source: Nat Commun. 2024 Jan 25;15:752. doi: 10.1038/s41467-024-45083-8 (PMC10810852; doi:10.1038/s41467-024-45083-8)
Supplement: Supplementary file 3 — Reporting Summary [file 41467_2024_45083_MOESM3_ESM.pdf]

nature portfolio

Corresponding author(s): Emmanuella Guenova, Wolfram Hoezenecker

Last updated by author(s): 2023.12.27

Reporting Summary

Nature Portfolio wishes to improve the reproducibility of the work that we publish. This form provides structure for consistency and transparency in reporting. For further information on Nature Portfolio policies, see our Editorial Policies and the Editorial Policy Checklist.

Statistics

For all statistical analyses, confirm that the following items are present in the figure legend, table legend, main text, or Methods section.

n/a	Confirmed	
☐	☒	The exact sample size (*n*) for each experimental group/condition, given as a discrete number and unit of measurement
☐	☒	A statement on whether measurements were taken from distinct samples or whether the same sample was measured repeatedly
☐	☒	The statistical test(s) used AND whether they are one- or two-sided<br>*Only common tests should be described solely by name; describe more complex techniques in the Methods section.*
☒	☐	A description of all covariates tested
☒	☐	A description of any assumptions or corrections, such as tests of normality and adjustment for multiple comparisons
☐	☒	A full description of the statistical parameters including central tendency (e.g. means) or other basic estimates (e.g. regression coefficient) AND variation (e.g. standard deviation) or associated estimates of uncertainty (e.g. confidence intervals)
☐	☒	For null hypothesis testing, the test statistic (e.g. *F*, *t*, *r*) with confidence intervals, effect sizes, degrees of freedom and *P* value noted<br>*Give P values as exact values whenever suitable.*
☒	☐	For Bayesian analysis, information on the choice of priors and Markov chain Monte Carlo settings
☒	☐	For hierarchical and complex designs, identification of the appropriate level for tests and full reporting of outcomes
☒	☐	Estimates of effect sizes (e.g. Cohen's *d*, Pearson's *r*), indicating how they were calculated

Our web collection on statistics for biologists contains articles on many of the points above.

Software and code

Policy information about availability of computer code

Data collection	Becton Dickinson FACSCantoTM and LSRFortessa™ instruments were used for FACS analysis; Capture and processing of single skin T cells was performed using the Fluidigm C1 Autoprep system; The single-cell cDNA products from each cell were then used to prepare Illumina sequencing libraries and sequenced as paired-end 150-base reads on the Illumina HiSeq 4000 platform. Western blots were visualized and imaged by the VILBER FUSION imaging system. Real-time PCR was applied using the QuantStudio™ 12K Flex Real-Time PCR System. Fluorescent images were obtained using the Cytation 3 cell imaging multi-mode reader. PerkinElmer IVIS Spectrum In Vivo Imaging System was used for bioluminescence live imagings. The nanoString mRNA expression was performed on the nCounter platform.
Data analysis	Flow cytometry data were analyzed using FCS Express 7 Flow Cytometry RUO (De Novo Software). Graphs and statistics were performed by GraphPad Prism v9.1.0. Quality control of single cell RNA-seq was performed using FastQC v0.11.7, and the adaptors and low quality bases with a Phred quality score <20 were trimmed from the ends of the reads using Trim Galore v0.4.4. The TraCeR system was used to reconstruct TCR recombinants independently for every cell, and gene expressions for cells with an identified TCR were quantified with Kallisto v0.45.0. The gene-expression data were normalized with Scater v1.10.1 and Scran v1.10.2. The TCRβ beta chain variable distribution plot was generated using mosaicplot. The gene expression of top genes on real-time stage was generated by SuperPlots. Seurat (v3.1) was used to scale and normalize gene-expression data for clustering and differential gene-expression analysis. Gene set enrichment analysis was conducted using gprofiler2. The nanoString data were normalized and analyzed using nSolver 4.0. The expression of selected genes was used to generate the heatmap using R v4.3.0.

For manuscripts utilizing custom algorithms or software that are central to the research but not yet described in published literature, software must be made available to editors and reviewers. We strongly encourage code deposition in a community repository (e.g. GitHub). See the Nature Portfolio guidelines for submitting code & software for further information.

Data

Policy information about availability of data

All manuscripts must include a data availability statement. This statement should provide the following information, where applicable:

- Accession codes, unique identifiers, or web links for publicly available datasets
- A description of any restrictions on data availability
- For clinical datasets or third party data, please ensure that the statement adheres to our policy

The single-cell RNA seq data of MF patients generated in this study have been deposited in the GEO database under accession code GSE224449 [https://www.ncbi.nlm.nih.gov/geo/query/acc.cgi?acc=GSE224449]. The single-cell RNA seq publicly available data of healthy controls, MF patients, AD patients, BCC patients and CBCL patients used in this study are available in the GEO database under accession code GSE173205 [https://www.ncbi.nlm.nih.gov/geo/query/acc.cgi?acc=GSE173205], GSE165623 [https://www.ncbi.nlm.nih.gov/geo/query/acc.cgi?acc=GSE165623], GSE222840 [https://www.ncbi.nlm.nih.gov/geo/query/acc.cgi?acc=GSE222840], GSE181907 [https://www.ncbi.nlm.nih.gov/geo/query/acc.cgi?acc=GSE181907] and GSE173820 [https://www.ncbi.nlm.nih.gov/geo/query/acc.cgi?acc=GSE173820].The neural network logistic regression (NN-log-reg) model is available online with publication at https://github.com/suskim/NN-log-reg. The single-cell RNA seq analysis is available online with publication at https://github.com/GuenovaLab/TumorLymphocytes

Research involving human participants, their data, or biological material

Policy information about studies with human participants or human data. See also policy information about sex, gender (identity/presentation), and sexual orientation and race, ethnicity and racism.

Reporting on sex and gender	The patients who met diagnostic criteria for early-stage MF and stage classification were recruited in this study, regardless the gender. Data on gender are available in the section "Population characteristics" below.
Reporting on race, ethnicity, or other socially relevant groupings	No socially consctructed or relevant categorisation variables were used in this study.
Population characteristics	Sample ; Gender ; Age at sample acquisition (years) ; Date of sample acquisition ; Sample type ; Disease stage at sample acquisition ; TNMB at sample acquisition ; First appearance of skin problem ; First diagnosis of CTCL ; Disease stage at 5 years' follow-up/death ; TNMB at 5 years' follow-up/death/SCT<br>1. MF028_BE ; Male ; 58 ; 2014-06 ; blood ; IA ; T1N0M0B0 ; 2012 ; 2012-08 ; IIB ; T3N0M0B0<br>2. MF028_BL ; Male ; 60 ; 2016-05 ; blood ; IIB ; T3N0M0B0 ; 2012 ; 2012-08 ; IIB ; T3N0M0B0<br>3. MF028_BLC ; Male ; 60 ; 2016-06 ; blood ; IIB ; T3N0M0B0 ; 2012 ; 2012-08 ; IIB ; T3N0M0B0<br>4. MF028_SE ; Male ; 57 ; 2013-05 ; skin ; IA ; T1N0M0B0 ; 2012 ; 2012-08 ; IIB ; T3N0M0B0<br>5. MF028_SL ; Male ; 60 ; 2016-05 ; skin ; IIB ; T3N0M0B0 ; 2012 ; 2012-08 ; IIB ; T3N0M0B0<br>6. MF032_BE ; Male ; 65 ; 2014-02 ; blood ; IIA ; T2N1M0B1 ; 2003 ; 2011-05 ; IVA1 ; T3N0M0B2<br>7. MF032_BL ; Male ; 67 ; 2016-09 ; blood ; IIB ; T3N1M0B1 ; 2003 ; 2011-05 ; IVA1 ; T3N0M0B2<br>8. MF032_SE ; Male ; 66 ; 2014-09 ; skin ; IIA ; T2N1M0B1 ; 2003 ; 2011-05 ; IVA1 ; T3N0M0B2<br>9. MF032_SL ; Male ; 68 ; 2016-12 ; skin ; IIB ; T3N1M0B1 ; 2003 ; 2011-05 ; IVA1 ; T3N0M0B2<br>10. MF030_BE ; Male ; 68 ; 2013-06 ; blood ; IA ; T1N0M0B0 ; 2011 ; 2011 ; IVB ; T3N0M1B2<br>11. MF030_BL ; Male ; 71 ; 2016-11 ; blood ; IIB ; T3N0M0B0 ; 2011 ; 2011 ; IVB ; T3N0M1B2<br>12. MF030_SE ; Male ; 68 ; 2013-06 ; skin ; IA ; T1N0M0B0 ; 2011 ; 2011 ; IVB ; T3N0M1B2<br>13. MF030_SL ; Male ; 71 ; 2016-09 ; skin ; IIB ; T3N0M0B0 ; 2011 ; 2011 ; IVB ; T3N0M1B2<br>14. MF029_S ; Female ; 71 ; 2013-03 ; skin ; IB ; T2N0M0B0 ; 2006 ; 2009 ; IVB ; T3N1B0bM1<br>15. MF040_S ; Male ; 66 ; 2014-02 ; skin ; IA ; T1N0M0B0 ; 2011 ; 2013 ; IIB ; T3N0B0M0<br>16. MF035_S ; Female ; 65 ; 2013-09 ; skin ; IB ; T2N0M0B0 ; 2003 ; 2011-09 ; IVB ; T3N0M1B0<br>17. MF201_S ; Female ; 70 ; 2014-03 ; skin ; IIA ; T2N1M0B1 ; 2008 ; 2010-07 ; IVA1 ; T4N1M0B2<br>18. MF200_S ; Male ; 72 ; 2014-02 ; skin ; IIA ; T2N1M0B1 ; 2011 ; 2013-10 ; IVA1 ; T4N0M0B2<br>19. MF202_S ; Male ; 76 ; 2013-06 ; skin ; IIA ; T2N2M0B1 ; 2010 ; 2010-12 ; IVA2 ; T4N3M0B2<br>20. MF053_S ; Male ; 54 ; 2014-10 ; skin ; IB ; T2N0M0B0 ; 2014 ; 2014-08 ; IVA1 ; T3N2M0B2<br>21. MF056_S ; Male ; 48 ; 2017-02 ; skin ; IB ; T2N0M0B0 ; 2005 ; 2011 ; IB ; T2N0M0B0<br>22. MF057_S ; Female ; 66 ; 2017-01 ; skin ; IB ; T2N0M0B0 ; 2011 ; 2016-12 ; IB ; T2N0M0B0<br>23. MF042_S ; Male ; 77 ; 2013-03 ; skin ; IB ; T2N0M0B0 ; 2004 ; 2013 ; IIB ; T3N0M0B0<br>24. MF046_S ; Female ; 73 ; 2015-10 ; skin ; IB ; T2N0M0B0 ; 2009 ; 2015-03 ; IB ; T2N0M0B0
Recruitment	All patients in this study met diagnostic criteria for early-stage MF according to the tumor/node/metastasis system and stage classification. Only patients from whom enough bio-banked research material was available could be included in the analysis. All consecutive patients fulfilling the criteria were considered.
Ethics oversight	All experiments in this study were conducted in accordance with the principles of the Declaration of Helsinki, and the study design was approved by the Institutional Review Boards of the Universities of Zurich and Lausanne. All of the patients provided written informed consent for the study.

Note that full information on the approval of the study protocol must also be provided in the manuscript.

Field-specific reporting

Please select the one below that is the best fit for your research. If you are not sure, read the appropriate sections before making your selection.

☒ Life sciences ☐ Behavioural & social sciences ☐ Ecological, evolutionary & environmental sciences

For a reference copy of the document with all sections, see nature.com/documents/nr-reporting-summary-flat.pdf

Life sciences study design

All studies must disclose on these points even when the disclosure is negative.

Sample size	Sample size was determined by the availability of the samples. We included as many available samples as possible, but we tried to use at least three samples to be able to derive statistical analysis.
Data exclusions	The low-quality cells and the non-TCR–reconstructed cells without reconstructed α and β chains from each sample were excluded.
Replication	1. Antibody-dependent cellular cytotoxicity (ADCC) experiments were performed at least three times for each patient.<br>2. siRNA transfection and real-time PCR were perfomred three times with similar results.<br>3. Due to sample availability (patient samples), scRNA-seq, FACS analysis, Western blot, NanoString were performed once per sample. However, we have at least three samples from three different patients to confirm the results.<br>4. The mouse experiments were repeated twice per condition with similar results.
Randomization	No randomization was performed. It's not applicable as we included the patients based on the diagnosis.
Blinding	For the human (patients) studies, investigators performed, acquired and analysis the experiments were not blinded since the results linked to the diseases of the patients. For the mouse experiments, investigators were not blinded as the investigators needed to well-document the treatments according the authorized rules. The investigators were blinded for tumor size measurement and In vivo imaging.

Reporting for specific materials, systems and methods

We require information from authors about some types of materials, experimental systems and methods used in many studies. Here, indicate whether each material, system or method listed is relevant to your study. If you are not sure if a list item applies to your research, read the appropriate section before selecting a response.

Materials & experimental systems

n/a	Involved in the study	
☐	☒	Antibodies
☐	☒	Eukaryotic cell lines
☒	☐	Palaeontology and archaeology
☐	☒	Animals and other organisms
☒	☐	Clinical data
☒	☐	Dual use research of concern
☒	☐	Plants

Methods

n/a	Involved in the study	
☒	☐	ChIP-seq
☐	☒	Flow cytometry
☒	☐	MRI-based neuroimaging

Antibodies

Antibodies used	For in vitro and in vivo experiment:<br>Anti-CD20-mAb (a chimeric human IgG1; MabThera® 10 mg/ml, Roche Pharma [Schweiz] AG) and anti-CD52-mAb (human IgG1; Lemtrada® 30 mg/ml, Sanofi-Aventis). Anti-CCR4-mAb (human IgG1; Gly-007CL) was purchased from Creative Biolabs. Anti-KIR-mAb, a fully human IgG4 that targets KIR2D (lirilumab; HY-P99208-1MG), was ordered from MedChemExpress. Commercially available monoclonal blocking antibody against human MHC-I (clone B9.12.1; Beckman Coulter, Krefeld, Germany), along with its F(ab')2 fragment and isotype control (mouse IgG2a; BioLegend), were used at a concentration of 10 μg/ml for the MHC-I blockade experiments. Anti-MHC-I F(ab')2 fragments were prepared with the Pierce F(ab')2 Micro Preparation Kit (Thermo Fisher, 44688) and verified by nonreducing sodium dodecyl sulfate polyacrylamide gel electrophoresis (SDS-PAGE). Monoclonal mouse IgG2a antibody against human CD20, anti-hCD20-mIgG2a (hcd20-mab10, InvivoGen), which features the constant region of the mouse IgG2a isotype and the variable region of anti-CD20-mAb, was used as a therapeutic antibody in in vitro and in vivo mouse experiments. For in vitro experiments, anti-hCD20-mIgG2a was used at a concentration of 10 μg/ml; for in vivo experiments, 250 μg of anti-hCD20-mIgG2a was administered intraperitoneally (i.p.) once weekly for three weeks. An equal amount of mouse IgG2a isotype control (BioLegend, 401504) was used in control groups. The F(ab')2 fragments of mouse monoclonal antibody against mouse Ly49 C and Ly49 I (mouse IgG2a, clone 5E6; BD Biosciences) and isotype mouse IgG2a (clone G155-178, BD Biosciences) were also used in vivo. Anti-Ly49 C/I F (ab')2 was used in vitro as a blocking antibody at a concentration of 10 μg/ml, and 200 μg of blocking anti-Ly49 C/I F(ab')2 was administered i.p. twice weekly for three weeks in in vivo experiments. An equal amount of mouse IgG2a isotype F(ab')2 was used in control groups.

For flow cytometry:
human: monoclonal antibodies against human CD3 (clone BW264/56; label PerCP; Miltenyi Biotec, 130-096-910, 130-113-131), CD3 (clone: SK7; label FITC; Invitrogen, 11-0036-42), CD3 (clone: HIT3a; label PerCP/Cyanine5.5; Biolegend, 300328), CD4 (clone VIT4; label APC-Vio770; Miltenyi Biotec, 130-098-153), CD4 (clone: RPA-T4; label APC/Cyanine7; Biolegend, 300518), CD8 (clone BW135/80; label PE-Cy7; Miltenyi Biotec, 130-096-556), CD8 (clone: SK1; label PE/Cyanine7; Biolegend, 344712), CD16 (clone VEP13; label APC; Miltenyi Biotec, 130-091-246), CD20 (clone LT20; label FTIC; Miltenyi Biotec, 130-098-081), CD45 (clone 5B1; label APC-Vio770; Miltenyi Biotec, 130-096-609), CD45 (clone HI30; label PerCP-Cyanine5.5; eBioscience, 45-0459-41), CD56 (clone AF12-7H3; label PE-Vio770; Miltenyi Biotec, 130-096-831), TCR Vβ (clone ZOE; label PE; Beckman Coulter), HLA-A,B,C (clone REA230; label FITC; Miltenyi Biotec, 130-101-447), HLA-A,B,C (clone: W6/32; label APC; Biolegend, 311410) and HLA-E (clone: 3D12; label Brilliant Violet 421; Biolegend, 342612). Isotype-matched negative control antibodies were used to set the gates for positive staining. Vβ clonal T-cell populations were assessed by flow cytometry using the IOTest® Beta Mark TCR Vβ Repertoire Kit (Beckman Coulter, IM3497).
mouse: monoclonal antibodies against mouse CD3 (clone 17A2; label PerCP; BioLegend , 100217), CD4 (clone RM4-5; label APC-Vio770; BioLegend, 100525), CD8 (clone 53-6.7; label PE-Vio770; BioLegend, 100721), CD45 (clone 30-F11; label APC; BioLegend, 103111), H-2Kb (clone AF6-88.5; label PE; BioLegend, 116507) and APC-conjugated anti-hCD20-mIgG2a (APC conjugation kit; Abcam, ab201807)

For Western blots:
The membranes were incubated separately with primary antibodies: anti-HLA-A (1:1000 dilution; ThermoFisher, PA5-29911), anti-HLA-B (1:3000 dilution; abcam, ab193415), anti-HLA-C (1:3000 dilution; abcam, ab193432), anti-HLA-E (1:1000 dilution; abcam, ab300553), anti-IFITM1 (1:1000 dilution; ThermoFisher, MA5-35972), anti-IFITM2 (1:1000 dilution; ThermoFisher, MA5-27503), anti-IL32 (1:500 dilution; ThermoFisher, PA5-119847) and anti-actin (1:5000 dilution; BD Biosciences, 612657). After incubation with primary antibody, the membranes were incubated with secondary antibodies: either anti-mouse IgG-HRP (1:5000 dilution) (Thermo Fisher, 31430) or anti-rabbit IgG-HRP (1:5000 dilution) (Thermo Fisher, A16023), depending on the source of the primary antibody.

Validation

Anti-KIR-mAb, a fully human IgG4 that targets KIR2D (lirilumab; HY-P99208-1MG)
https://www.medchemexpress.com/lirilumab.html

Anti-human MHC-I (clone B9.12.1; Beckman Coulter, Krefeld, Germany)
https://www.beckman.com/reagents/coulter-flow-cytometry/antibodies-and-kits/single-color-antibodies/hla-abc/im1838u

anti-hCD20-mIgG2a (hcd20-mab10, InvivoGen)
https://www.invivogen.com/anti-human-cd20-mouse-igg2a-rituximab

mouse IgG2a isotype control (BioLegend, 401504)
https://www.labome.com/product/BioLegend/401504.html

anti-mouse Ly49 C and Ly49 I (mouse IgG2a, clone 5E6; BD Biosciences)
https://www.bdbiosciences.com/en-us/products/reagents/flow-cytometry-reagents/research-reagents/single-color-antibodies-ruo/purified-na-le-mouse-anti-mouse-ly-49c-and-ly-49i.553273

isotype mouse IgG2a (clone G155-178, BD Biosciences)
https://www.bdbiosciences.com/en-us/products/reagents/flow-cytometry-reagents/research-reagents/single-color-antibodies-ruo/purified-mouse-igg2a-isotype-control.553454

anti-human CD3 (clone BW264/56; label PerCP; Miltenyi Biotec, 130-113-131)
https://www.miltenyibiotec.com/CH-en/products/cd3-antibody-anti-human-bw264-56.html#conjugate=viogreen:size=100-tests-in-200-ul

anti-human CD3 (clone: SK7; label FITC; Invitrogen, 11-0036-42)
https://www.thermofisher.com/antibody/product/CD3-Antibody-clone-SK7-Monoclonal/11-0036-42

anti-human CD3 (clone: HIT3a; label PerCP/Cyanine5.5; Biolegend, 300328)
https://www.biolegend.com/de-at/products/percp-cyanine5-5-anti-human-cd3-antibody-5613

anti-human CD4 (clone VIT4; label APC-Vio770; Miltenyi Biotec, 130-098-153)
https://www.miltenyibiotec.com/CH-en/products/cd4-antibody-anti-human-vit4.html#conjugate=apc-vio-770:size=30-tests-in-60-ul

anti-human CD4 (clone: RPA-T4; label APC/Cyanine7; Biolegend, 300518)
https://www.biolegend.com/de-at/products/apc-cyanine7-anti-human-cd4-antibody-1933

anti-human CD8 (clone BW135/80; label PE-Cy7; Miltenyi Biotec, 130-096-556)
https://www.miltenyibiotec.com/CH-en/products/cd8-antibody-anti-human-bw135-80.html#conjugate=viogreen:size=100-tests-in-200-ul

anti-human CD8 (clone: SK1; label PE/Cyanine7; Biolegend, 344712)
https://www.biolegend.com/de-at/products/pe-cyanine7-anti-human-cd8-antibody-6390

anti-human CD16 (clone VEP13; label APC; Miltenyi Biotec, 130-091-246)
https://www.miltenyibiotec.com/CH-en/products/cd16-antibody-anti-human-vep13.html#conjugate=biotin:size=100-tests-in-200-ul

anti-human CD20 (clone LT20; label FTIC; Miltenyi Biotec, 130-098-081)

https://www.miltenyibiotec.com/CH-en/products/cd20-antibody-anti-human-lt20.html

anti-human CD45 (clone 5B1; label APC-Vio770; Miltenyi Biotec, 130-096-609)
https://www.miltenyibiotec.com/CH-en/products/cd45-antibody-anti-human-5b1.html#conjugate=viogreen:size=100-tests-in-200-ul

anti-human CD45 (clone HI30; label PerCP-Cyanine5.5; eBioscience, 45-0459-41)
https://www.thermofisher.com/antibody/product/CD45-Antibody-clone-HI30-Monoclonal/45-0459-42

anti-human CD56 (clone AF12-7H3; label PE-Vio770; Miltenyi Biotec, 130-096-831)
https://www.miltenyibiotec.com/CH-en/products/cd56-antibody-anti-human-af12-7h3.html#conjugate=vio-bright-fitc:size=100-tests-in-200-ul

anti-human TCR Vβ (clone ZOE; label PE; Beckman Coulter)
https://www.beckman.com/reagents/coulter-flow-cytometry/antibodies-and-kits/single-color-antibodies/tcr-vb-2/im2213

anti-human HLA-A,B,C (clone REA230; label FITC; Miltenyi Biotec, 130-101-447)
https://www.miltenyibiotec.com/CH-en/search/REA230

anti-human HLA-A,B,C (clone: W6/32; label APC; Biolegend, 311410)
https://www.biolegend.com/de-at/products/apc-anti-human-hla-a-b-c-antibody-1870

anti-human HLA-E (clone: 3D12; label Brilliant Violet 421; Biolegend, 342612)
https://www.biolegend.com/de-at/products/brilliant-violet-421-anti-human-hla-e-antibody-15290

Vβ clonal T-cell populations were assessed by flow cytometry using the IOTest® Beta Mark TCR Vβ Repertoire Kit (Beckman Coulter, IM3497)
https://www.beckman.com/reagents/coulter-flow-cytometry/antibodies-and-kits/clinical-research-systems-and-kits/im3497

Anti-mouse CD3 (clone 17A2; label PerCP/Cyanine5.5; BioLegend , 100217)
https://www.biolegend.com/de-at/products/percp-cyanine5-5-anti-mouse-cd3-antibody-5596

Anti-mouse CD4 (clone RM4-5; label APC-Vio770; BioLegend, 100525)
https://www.biolegend.com/de-at/products/apc-cyanine7-anti-mouse-cd4-antibody-1937

Anti-mouse CD8 (clone 53-6.7; label PE-Vio770; BioLegend, 100721)
https://www.biolegend.com/de-at/products/pe-cyanine7-anti-mouse-cd8a-antibody-1906

Anti-mouse CD45 (clone 30-F11; label APC; BioLegend, 103111)
https://www.biolegend.com/de-at/products/apc-anti-mouse-cd45-antibody-97

Anti-mouse H-2Kb (clone AF6-88.5; label PE; BioLegend, 116507)
https://www.biolegend.com/de-at/products/pe-anti-mouse-h-2kb-antibody-1749

Eukaryotic cell lines

Policy information about cell lines and Sex and Gender in Research

Cell line source(s)	Raji (ATCC-CCL-86, ATCC); Ramos (RA1, ATCC-CRL-1596, ATCC); My-La CD4+ (ECACC, catalog no. 95051032, Merck); EL4 (ATCC TIB-39, ATCC); EL4-hCD20 (Clone 1E12A5) was provided by Dr Jeanette Leusen (Laboratory for Translational Immunology, UMC Utrecht, the Netherlands).
Authentication	None of cell lines used were authenticated in the lab; however, all cell lines were purchased directly and authenticated by the suppliers. The gene-modified EL4-hCD20 (Clone 1E12A5) were authenticated by morphology and confirmed with the expression of mouse CD3, human CD20, luciferase and green fluorescent protein.
Mycoplasma contamination	All of the cell lines were tested negative for mycoplasma contamination
Commonly misidentified lines (See ICLAC register)	No cell line used in the study was found in the databases of commonly misidentified cell lines.

Animals and other research organisms

Policy information about studies involving animals; ARRIVE guidelines recommended for reporting animal research, and Sex and Gender in Research

Laboratory animals	Wild-type C57BL/6 mice were purchased from Envigo. Age-matched (6–12 weeks) female animals were used throughout experiments. Animal experiments were approved by the Swiss regulatory authorities and all mice were kept in accordance with regulations from the authorized Laboratory Animal Services Center. All mice housed in conventional animal facility of University Hospital of Zurich were kept in individually ventilated cages, between 19-23 °C with 45-65% humidity and a 12-hour dark/light cycle.

Wild animals	No wild animals were used in this study.
Reporting on sex	Female C57BL/6 mice were used for EL4 mouse model.
Field-collected samples	No field-collected samples were used in this study.
Ethics oversight	Animal experiments were approved by the Swiss regulatory authorities

Note that full information on the approval of the study protocol must also be provided in the manuscript.

Flow Cytometry

Plots

Confirm that:

- [x] The axis labels state the marker and fluorochrome used (e.g. CD4-FITC).
- [x] The axis scales are clearly visible. Include numbers along axes only for bottom left plot of group (a 'group' is an analysis of identical markers).
- [x] All plots are contour plots with outliers or pseudocolor plots.
- [x] A numerical value for number of cells or percentage (with statistics) is provided.

Methodology

Sample preparation	For flow cytometry analysis, cells were collected, washed and resuspended in ice-cold magnetic-activated cell sorting (MACS) buffer (phosphate-buffered saline [PBS, pH 7.2], 0.5% bovine serum albumin, and 2 mM ethylenediaminetetraacetic acid) and fluorescent-conjugated antibodies for 20 minutes on ice, followed by two washes with MACS buffer.
Instrument	Becton Dickinson FACSCantoTM and LSRFortessa™
Software	FCS Express 7 Flow Cytometry RUO (De Novo Software)
Cell population abundance	We ensured that at least 10,000 cells were counted for each samples.
Gating strategy	We gated on single-cell lymphocyte population and then analyzed the positive and negative staining cells.

- [x] Tick this box to confirm that a figure exemplifying the gating strategy is provided in the Supplementary Information.
